# Reflections on Some Urinary Troubles, Such as Gravel and Enlarged Prostate, and Other Incidental Remarks

**Published:** 1885-11

**Authors:** B. Ehrman

**Affiliations:** Cincinnati


### REFLECTIONS ON SOME URINARY TROUBLES, SUCH AS GRAVEL AND ENLARGED PROSTATE, AND OTHER INCIDENTAL REMARKS.

B. Ehrman, M. D., Cincinnati.

We have lost in this community some of our best citizens, with these complaints, who might have lived a good many years longer if properly attended to. But, being regarded as surgical diseases, patients are prone to confide in surgeons until they relieve them of all aches or pains.

The readers of this journal as well as the *Advance* will recollect some controversy about the curability or non-curability of this disease of old age, enlarged prostate. The young doctor (although a graduate of a homœopathic college), being entirely guided by Gross as to diagnosis and prognosis, ridiculed the pretension of homœopaths to cure the same.

The writer of these lines, having himself suffered lately for five or six weeks severely with this disease, and being now convalescent for some time and in a fair way of a complete cure—although he is the senior by twelve years of most of those that have died within his knowledge for the last five or six years and in consideration of the fact that even our homœopathic physicians will doubt and despair—feels it his duty to testify to the curability of the disease under consideration for the benefit of suffering humanity.

Now let me relate the history of one such case that was partially under my care, and who eventually fell a victim to bungling surgery :

This patient passed occasionally some small gravel for many years—maybe once in three, four, or six months—without much inconvenience. Being otherwise in good health and robust constitution, he was not disposed to become a regular patient for the eradication of this gravel dyscrasia, but would simply take a prescription occasionally whenever he thought best.

In consequence of one of his neighbors being operated on for stone in the bladder, he felt some apprehension that he might be in the same condition, and concluded to have himself examined. The surgeon found no stone nor any other lesion.

Some weeks after, he called at my office and related to me what I have just stated, and jocosely remarked that "the surgeon gave him with his instrument the clap," for which he now engaged my services, after this surgeon had failed to remedy the trouble which he had induced by his instrument.

I had prescribed but a few times for this urethritis in my office, and mostly by proxy, when the young doctor came to my office to inform me that he, in consultation with another surgeon (a homœopath), had examined my patient and found enlarged prostrate and *no gravel* at all. The young doctor, a near relative and living in the same house, now prescribed, but after five months' treatment, during the best season of the year, had failed to do him any good, and I was recalled. After three weeks' treatment I received a letter from him (for I visited him only when requested to do so), stating that he was a good deal better, and that he had to use the catheter only two or three times during the night, whereas he had to use it very often before.

But, to make the matter short, the young doctor used every opportunity to get him again under his care, merely to palliate this enlarged prostate, as a cure was impossible according to the authority on which he relied. After treating him for a few months for that purpose, he died suddenly and unexpectedly. Now we come to the most remarkable part of this story. The post-mortem examination revealed the following facts: The prostrate had sloughed away, and the bladder contained twenty-three calculi, some as large as buckeyes and others smaller.

Failures as well as cures are very useful if the proper lessons are deduced from them. Therefore:

Remark 1. It is my candid opinion that if the patient had never seen that first surgeon, he would be alive yet, and lived for many years more as comfortable as he had done before. There would have been no urethritis for me to treat, and there would not have been an opportunity for the young doctor with his homœopathic surgeon to step in clandestinely to take the patient out of my hands, just at the beginning of my treatment for this urethritis, induced by the first surgeon, under the plea that I treated him for a wrong disease, gravel dyscrasia.

Remark 2. When I was recalled to attend the patient again, he told me incidentally that a neighbor had handed him a certain root, which he said had the power to dissolve stone in the bladder when used as a tea. He concluded to inject an infusion of it into his bladder, on the supposition that it would be more efficacious in this way than as a drink merely. This he *did* all on his own responsibility and without my knowledge, while under my care, in the first weeks of my treatment. A few days after this the two savants came to examine the patient, and as they found no gravel disease, they found their efforts well rewarded by discovering the prostate enlarged, as they said. Now, supposing it was larger than usual with a man of his age, and suffering as he did for many weeks, the question now comes up forcibly: If that root had any such power as to dissolve calculi, it must certainly be irritating, not only to the parts injected, but also to the surroundings. The prostate gland would naturally be larger, just as the parotid glands enlarge in malignant diphtheria or scarlatina, by sympathy.

Remark 3. Whenever there is any urinary disease, everybody will recommend some natural mineral water, such as Bethesda water, as if it were a cure for all cases. So this patient was advised by his doctors to drink of it as much as he could, day and night, and have the urine drawn by the catheter altogether.

According to my experience and observation, I regard this Bethesda water homœopathic to gravel dyscrasia, in so far as it favors the formation and accumulation of gravel in the bladder; and, when employed or administered homœopathically, might, no doubt, do good.

But to do as the patient was advised by the two savants—to drink plenty of it day and night, and have all the urine drawn by the catheter, thereby preventing the passing off of small particles, and disregarding the very important element of diet in this disease—was the height of folly, and nothing but a fatal end could be expected.

